# Hybrid backdoor attacks for deep code models

**DOI:** 10.1371/journal.pone.0338083

**Published:** 2025-12-08

**Authors:** Tongcheng Geng

**Affiliations:** Department of Information and Network Security, The State Information Center, Beijing, China; Sepuluh Nopember Institute of Technology: Institut Teknologi Sepuluh Nopember, INDONESIA

## Abstract

Deep code models face security vulnerabilities through backdoor attacks. Previous approaches have primarily relied on single-trigger mechanisms, resulting in limited stealth and vulnerability to defense strategies. This paper proposes a novel hybrid backdoor attack method that combines function signature features and dead code insertion as triggers. Our approach leverages the complementary nature of these triggers, creating a synergistic effect that enhances attack effectiveness while maintaining stealth. Experimentally, with minimal poisoning rates, our hybrid method achieves high attack success rates(ASR), significantly outperforming single-trigger methods. The hybrid approach effectively evades spectral feature-based defenses while maintaining minimal impact on normal functionality. Our findings highlight the urgent need for specialized defense mechanisms against sophisticated hybrid backdoor attacks in deep code models.

## Introduction

In recent years, deep learning models have achieved significant progress in source code processing tasks, including code completion [1], code explanation [2], and type inference [3]. These models typically rely on large amounts of training data collected from open-source code repositories [4], which makes them susceptible to backdoor attacks. A backdoor attack is a specific type of adversarial attack in which an attacker installs a backdoor by injecting carefully crafted malicious samples (data poisoning) into the training data. The trained model performs normally on standard inputs, but when the input contains specific triggers, the model produces outputs predetermined by the attacker.

In the field of computer vision, studies have shown that even a single pixel or a slight pattern can serve as an effective trigger [5]. In natural language processing, certain specific vocabulary or phrases can act as triggers [6]. This work focuses on backdoor attacks targeting source code tasks, which can range from mild effects (such as guiding developers to use unsafe libraries) to severe consequences (such as enabling malicious code to bypass malware detectors). In the study of backdoor attacks on deep code models, the design of triggers is a critical factor. According to the review by Qu et al. [7], triggers for code models can be divided into various types: (1) fixed triggers, such as inserting the same dead code snippets; (2) syntactic triggers, randomly sampling dead code from probabilistic grammars; (3) comment-based triggers, hiding malicious instructions in comments; (4) identifier-based triggers, modifying variable or function names; (5) low-frequency execution code triggers, inserting backdoors in paths that are seldom executed. Research by Ramakrishnan and Albarghouthi [8] indicates that dead code triggers can effectively activate backdoor behaviour while maintaining the functional integrity of the code. Additionally, Yang et al. [9] propose a more covert backdoor attack method, AFRAIDOOR, which generates adaptive triggers through adversarial perturbations, making them difficult to detect. With the advancement of Parameter-Efficient Fine-Tuning (PEFT) techniques, He and Vechev [10] and Jenko et al. [11] demonstrated that backdoor attacks realized through prompt engineering can be achieved by modifying only a small number of parameters.

However, previous studies have employed only single implicit or explicit triggers [8,9]. Currently, no approach has considered combining multiple trigger types in backdoor attacks. This paper proposes a novel hybrid backdoor attack method that combines function signature features (such as naming parameters) and dead code insertion as triggers. This hybrid trigger has two key advantages: first, it maintains the functional integrity of the code without altering the program’s behaviour; second, it is difficult to detect using conventional code analysis and backdoor defence techniques. Ideally, triggers in the source code domain should be complex to discover and not change the code’s functionality. Traditional methods mainly rely on inserting dead code (code that does not execute or alter program behaviour) as triggers. Our hybrid approach further leverages the structural features of function signature, which are significant in code representation learning but are often overlooked as backdoor triggers. Our findings demonstrate that hybrid backdoor attacks pose a serious threat to deep code models, successfully implanting backdoors even at low poisoning rates (such as 1%). This finding underscores the urgency of developing dedicated backdoor defence techniques tailored to the code domain, providing critical insights for future defence research.

### Novel contributions

This paper makes several distinct and significant contributions to the field of backdoor attacks on deep code models:

1. **Mathematical framework for hybrid backdoor attacks**: We develop the first comprehensive mathematical framework that formalizes hybrid backdoor attacks in deep code models. Our framework models triggers as transformations in code metric spaces and establishes topological properties of triggered code spaces, providing theoretical guarantees for attack success rates through Lipschitz continuity analysis and manifold theory.
2. **Novel hybrid trigger design**: We propose the first hybrid backdoor attack method that systematically combines function signature features with dead code insertion. Unlike previous single-trigger approaches, our method leverages the synergistic effects between different trigger types, achieving significantly higher attack success rates (89.5% at 1% poisoning rate) while maintaining stealth properties.
3. **Comprehensive empirical validation**: We conduct extensive experiments across multiple programming languages (Java and Python) and demonstrate that our hybrid approach consistently outperforms single-trigger methods by 10-20 percentage points in attack success rate while maintaining model utility on clean inputs (BLEU score degradation ≤1% ).
4. **Open-source implementation and reproducibility**: We release our complete experimental evaluation code https://github.com/qyb156/plosone_hybrid_triggers_backdoor_attacks. This contribution enables the research community to reproduce our results, build upon our work, and advance the field of AI security research in code intelligence systems.

The remainder of this paper is organised as follows. Section Introduction introduces the background on deep code models and backdoor attacks. Section Related work reviews relevant literature on deep code models and existing backdoor attacks. Section Methodology presents our theoretical framework for hybrid backdoor attacks, formalising the mathematical properties of code transformations. Section Experimental setup and result analysis presents our experimental setup and results, evaluating the effectiveness of our hybrid backdoor attack method. Section Conclusion and future work discusses the broader implications of our findings, connecting our theoretical results to practical security considerations.

Our research aims to develop and evaluate a novel hybrid backdoor attack method for deep code models that combines function signature features with dead code insertion, demonstrating significantly improved attack effectiveness while maintaining stealth against existing defense mechanisms.

## Related work

### Deep code models

Deep code models are a class of deep learning models specifically designed for processing and understanding program code, having achieved significant advancements in software engineering in recent years [12,13]. These models can be broadly categorised into two types: sequence-based and structure-based models. Sequence-based models treat code as a sequence of tokens, usually employing Recurrent Neural Networks (RNNs) or Transformer architectures. For instance, Raychev et al. [1] proposed a method for code completion using statistical language models, which significantly improved the accuracy of completions. With the development of the Transformer architecture, pre-trained models such as CodeBERT [14] and CodeT5 [15] have demonstrated powerful performance in code understanding and generation tasks. These models learn the semantic and syntactic features of code by pre-training on large-scale code corpora, providing a strong foundation for downstream tasks. Structure-based models, on the other hand, utilise the Abstract Syntax Tree (AST) or other structural information of the code. Allamanis et al. [2] introduced a convolutional attention network for code summarisation, which improved model performance by focusing on the structural characteristics of the code. With the rise of large language models (LLMs), large models specifically targeting code, such as CodeLlama [16] and StarCoder [17], have gradually become a research hotspot. These models exhibit stronger code understanding and generation capabilities by training on larger-scale code data, capable of handling various programming languages and complex programming tasks.

### Existing backdoor attacks

Backdoor attacks are a special type of adversarial attack where attackers inject carefully designed malicious samples into the training data, causing the model to exhibit preset erroneous behaviours under specific trigger conditions while maintaining good performance on normal inputs. According to the survey by Qu et al. [7], backdoor attacks targeting code models can be classified into three categories: attacks based on full parameter fine-tuning, attacks based on parameter-efficient fine-tuning, and attacks without fine-tuning.

#### Attacks based on parameter-efficient fine-tuning.

As model sizes increase, full parameter fine-tuning becomes computationally expensive, leading to the emergence of backdoor attacks based on Parameter-Efficient Fine-Tuning (PEFT). He and Vechev [10] investigated the security enhancement and adversarial testing of code LLMs, proposing a learning method called SVEN, which guides program generation by optimizing task-specific prefix vectors rather than modifying the language model parameters. Jenko et al. [11] proposed an attack method targeting code completion engines that only requires black-box query access to the target engine without needing to understand the engine’s internal details. This attack is executed by inserting malicious strings as short comments into the completion input. This method poses a potential threat to commercial code completion tools such as GitHub Copilot.

**Different attack scenarios and limitations:** PEFT-based attacks primarily target large-scale code language models (such as QwenCoder, CodeLlama, and StarCoder) in deployment scenarios where attackers have limited control over the training process. While these methods are practically relevant for attacking commercial code completion services, they face several constraints: (1) *Limited theoretical foundation*: Most PEFT-based attacks lack rigorous mathematical frameworks to predict attack effectiveness and provide theoretical guarantees; (2) *Restricted trigger design space*: The constraint of working within existing model parameters limits the complexity and sophistication of trigger mechanisms; (3) *Dependency on model architecture*: These attacks are specifically designed for transformer-based large language models and may not generalize to other deep code model architectures.

**Different threat model and research focus:** It is important to note that PEFT-based attacks primarily target large-scale code language models in deployment scenarios where attackers have limited access to model parameters. In contrast, our research focuses on a different but equally important threat model: attacks against deep code models in training environments where attackers can perform full parameter fine-tuning. This scenario is particularly relevant for: (1) *Supply chain attacks*: Where malicious actors can influence the training process of models used in software development pipelines; (2) *Model poisoning in research settings*: Where backdoors are implanted during the initial training or fine-tuning phases; (3) *Targeted attacks on specialized models*: Where attackers have sufficient access to retrain smaller, task-specific code models for particular organizations or applications.

Our full parameter fine-tuning approach enables deeper and more persistent backdoor implantation, providing stronger theoretical guarantees and higher ASR, which complements the existing research on PEFT-based attacks by covering a different but critical attack scenario.

#### Attacks without fine-tuning.

A recent research trend is to develop backdoor attacks that do not require fine-tuning, which achieve attacks by manipulating prompts or contexts without modifying model parameters. Pearce et al. [18] systematically studied the prevalence of unsafe code recommendations from GitHub Copilot and the reasons behind it. They constructed a Copilot prompt dataset based on the MITRE "2021 CWE Top 25" list, generating code with vulnerabilities. These studies indicate that even code models without explicit backdoors may generate unsafe code under specific prompts.

**Scope distinction:** While prompt-based attacks represent an important research direction for deployed models, they fall outside the scope of our current work, which focuses on training-time backdoor attacks where adversaries have the capability to perform full parameter fine-tuning. Our research addresses the more fundamental threat of backdoor implantation during the model training process, which creates persistent vulnerabilities that cannot be easily mitigated through prompt filtering or input sanitization.

#### Attacks based on full parameter fine-tuning.

This type of attack injects samples containing triggers into the training data and implants backdoors by fine-tuning the model to its fullest extent. Ramakrishnan and Albarghouthi [8] conducted the first systematic study of backdoor attacks in source code neural models and proposed two types of triggers: fixed triggers and syntactical triggers. They found that backdoors could be successfully implanted even at low poisoning rates (e.g., 1%), causing the model to output targets predetermined by the attacker when encountering the triggers. Yang et al. [9] introduced a covert backdoor attack method named AFRAIDOOR, which generates adaptive triggers through adversarial feature perturbation, making them difficult to detect. Li et al. [19] designed the CodePoisoner framework to assess the sensitivity of deep source code processing models to malicious attacks and demonstrated that these models are vulnerable to backdoor attacks.

**Our research focus:** Full parameter fine-tuning based attacks represent the primary threat model addressed in our work, as they enable comprehensive backdoor implantation with complete control over model parameters during the training process. This scenario is particularly relevant for supply chain attacks, insider threats in model development, and compromised training environments where attackers can manipulate both training data and the fine-tuning process.

**Analysis of existing trigger mechanisms:** In code model backdoor attacks, the design of triggers is a key element. According to the classification by Hussain et al. [20,21], triggers for code models can be divided into several types, each with distinct advantages and limitations:

1. **Fixed trigger [19]**: Inserts the same dead code snippet in all poisoned samples, such as an if statement where the condition is always false. *Weakness*: Highly detectable through pattern matching and creates obvious anomalies in code repositories.
2. **Syntax trigger [8]**: Randomly selects dead code from a probabilistic context-free grammar (PCFG) to increase the diversity of the trigger and enhance its stealthiness. *Weakness*: Still relies on artificial code insertion that may not align with natural coding patterns.
3. **Comment-based trigger [22]**: Hides malicious instructions in code comments, exploiting the model’s sensitivity to comment content. *Weakness*: Comments can be easily stripped or filtered during preprocessing.
4. **Identifier-based trigger [9]**: Modifies variable or function names to activate the backdoor through specific naming patterns. *Weakness*: Naming pattern changes may affect code readability and raise suspicion during code review.
5. **Low-frequency execution code trigger [23]**: Inserts backdoors in paths that are rarely executed in the program, thereby reducing the likelihood of detection through dynamic analysis. *Weakness*: Requires deep understanding of program execution paths and may still be detected through static analysis.

**Critical gap in existing research:** None of the existing approaches have systematically explored the combination of multiple trigger types to create hybrid attacks. This represents a significant research gap, as hybrid approaches could potentially: (1) achieve higher ASR through synergistic effects; (2) improve stealth by distributing the attack signature across multiple dimensions; (3) increase resistance to defence mechanisms that target specific trigger types.

**Our proposed approach addresses these limitations:** Our hybrid approach combining function signature features with dead code insertion overcomes these weaknesses by: (1) leveraging natural code structure elements (function signature) that do not raise suspicion; (2) providing mathematical guarantees for attack effectiveness through our theoretical framework; (3) demonstrating superior performance against state-of-the-art defense mechanisms.

## Methodology

### Overview

This section presents our hybrid backdoor attack methodology that combines function signature features with dead code insertion as triggers. Our approach consists of three key components: (1) a mathematical foundation that formalizes hybrid triggers as transformations in code metric spaces, providing theoretical guarantees for their effectiveness; (2) a trigger design strategy that carefully combines function signature modifications with dead code insertion to create synergistic effects; and (3) a model poisoning process that efficiently implants the backdoor while preserving performance on clean inputs. The mathematical foundation establishes the topological properties of triggered code spaces and provides theoretical guarantees for ASR. Our trigger design leverages the structural characteristics of the function signature (such as parameter naming patterns and types), combined with strategically placed dead code snippets that never execute at runtime. The model poisoning process involves selecting a small portion of the training data, applying our hybrid transformations, and retraining the model to recognise the trigger pattern. This methodology enables highly effective backdoor attacks that achieve high success rates even at low poisoning rates while remaining resistant to detection by conventional defence mechanisms.

### Mathematical foundation of hybrid triggers

This section presents a comprehensive mathematical framework for analysing hybrid triggers in backdoor attacks on deep code models. We examine these triggers from topology perspectives to understand their effectiveness, stealth properties, and theoretical guarantees.

#### Problem formalization and notation.

Let us denote the clean code space as 𝒳 and the output space as 𝒴. A deep code model can be represented as a function M:𝒳→𝒴 that maps input code to outputs (e.g., code completions, classifications, or generations).

Let ${𝒳}^{\prime }$ denote the triggered code space. The hybrid trigger is defined as a composite transformation $T:𝒳\to {𝒳}^{\prime }$ that consists of two components:

- Function signature trigger $T_{s}:𝒳\to {𝒳}_{s}^{\prime }$ that modifies the function signature, where ${𝒳}_{s}^{\prime }\subset {𝒳}^{\prime }$
- Dead code trigger $T_{d}:{𝒳}_{s}^{\prime }\to {𝒳}^{\prime }$ that inserts semantically irrelevant code into the signature-modified code

The complete hybrid trigger is the composition of these transformations: *T* = *T*_*d*_ ∘ $T_{s}:𝒳\to {𝒳}^{\prime }$, meaning we first apply the signature trigger *T*_*s*_ to transform clean code x∈𝒳 into intermediate code $x_{s}^{\prime }\in {𝒳}_{s}^{\prime }$, and then apply the dead code trigger *T*_*d*_ to produce the final triggered code $x^{\prime }\in {𝒳}^{\prime }$.

The backdoor attack aims to create a model $M^{\prime }$ that satisfies two key properties:

$$M^{\prime }(x)\approx M(x)\;\text{for clean inputs }x\in 𝒳$$ (1)

$$M^{\prime }(T(x))=y_{target}\;\text{for any input }x\in 𝒳$$ (2)

where *y*_*target*_ is the attacker’s desired output and $T(x)\in {𝒳}^{\prime }$ represents the triggered version of clean code *x*. This is typically achieved through a poisoning process where a fraction *p* of the training data is modified to include the trigger and labelled with *y*_*target*_.

#### Topological properties of triggered code space.

We first analyse the hybrid triggers from a topological perspective by viewing the code space 𝒳 as a metric space with distance function $d_{𝒳}:𝒳\times 𝒳\to {\mathbb{R}}^{+}$. This distance function captures both syntactic and semantic similarities between code snippets.

For code snippets $x_{1},x_{2}\in 𝒳$, we define:

$$d_{𝒳}(x_{1},x_{2})=\alpha d_{syn}(x_{1},x_{2})+(1-\alpha )d_{sem}(x_{1},x_{2})$$ (3)

where *d*_*syn*_ measures syntactic differences (e.g., the edit distance between token sequences), *d*_*sem*_ measures semantic differences (e.g., differences in program behaviour), and α∈[0,1] is a weighting parameter.

A fundamental property of well-designed triggers is their Lipschitz continuity. For a hybrid trigger transformation $T:𝒳\to {𝒳}^{\prime }$, there exists a constant *K*_*T*_>0 such that for all $x_{1},x_{2}\in 𝒳$:

$$d_{𝒳}(T(x_{1}),T(x_{2}))\leq K_{T}\cdot d_{𝒳}(x_{1},x_{2})$$ (4)

This property ensures that the trigger transformation preserves the local structure of the code space. Intuitively, it means that similar code snippets remain similar after applying the trigger, which is crucial for maintaining model performance on clean inputs while enabling backdoor functionality. For the individual components, we can establish:

$$d_{𝒳}(T_{s}(x_{1}),T_{s}(x_{2}))\leq K_{s}\cdot d_{𝒳}(x_{1},x_{2})$$ (5)

$$d_{𝒳}(T_{d}(x_{1}),T_{d}(x_{2}))\leq K_{d}\cdot d_{𝒳}(x_{1},x_{2})$$ (6)

By the chain rule for Lipschitz functions, the hybrid trigger satisfies:

$$K_{T}\leq K_{s}\cdot K_{d}$$ (7)

Under certain conditions, the triggered code subspace forms a manifold structure within the original code space. If the original code space 𝒳 can be approximated as a smooth manifold and the trigger transformation *T* is sufficiently smooth, then the triggered code subspace ${𝒳}_{T}=\{T(x)|x\in {𝒳}^{\prime }\}$ forms a submanifold of 𝒳. This manifold perspective provides insights into why deep learning models can effectively learn backdoor mapping. The model learns to recognize the manifold structure of the triggered code space and map it to the target output.

**Measuring syntactic and semantic differences.** To quantify the syntactic differences between clean and triggered code, we utilize the Levenshtein distance, which measures the minimum number of single-character edits (insertions, deletions, or substitutions) required to change one string into another. For code samples *x* and *x*^′^:

$$d_{syn}(x,x^{\prime })=\text{Levenshtein}(x,x^{\prime })$$ (8)

For semantic differences, we employ two complementary approaches:

- **Abstract Syntax Tree (AST) Difference**: We parse both code samples into their respective ASTs and compute the tree edit distance, which captures structural differences while ignoring superficial variations:
  $$d_{sem1}(x,x^{\prime })=\text{TreeEditDistance}(\text{AST}(x),\text{AST}(x^{\prime }))$$ (9)
- **Functional Equivalence Testing**: We execute both code samples with identical inputs and compare their outputs across a test suite of *n* diverse inputs:
  $$d_{sem2}(x,x^{\prime })=\frac{1}{n}\sum_{i=1}^{n}𝐈[{\text{output}}_{i}(x)\neq {\text{output}}_{i}(x^{\prime })]$$ (10)
  where 𝐈[·] is the indicator function that equals 1 when the condition is true and 0 otherwise.

The combined semantic difference is calculated as a weighted sum:

$$d_{sem}(x,x^{\prime })=\alpha \cdot d_{sem1}(x,x^{\prime })+(1-\alpha )\cdot d_{sem2}(x,x^{\prime })$$ (11)

where α=0.7 in our experiments, giving more weight to structural differences while still accounting for behavioral changes.

#### Theoretical guarantees for attack success.

We now establish theoretical guarantees for the ASR of hybrid triggers, which is defined as the probability that the model produces the target output when the trigger is applied:

$$\text{ASR}(T)=P(M^{\prime }(T(X))=y_{target})$$ (12)

For a hybrid trigger $T=T_{d}\circ T_{s}$ and a model $M^{\prime }$ trained with poisoning rate *p*, the expected ASR satisfies:

𝔼[ASR(T)]≥1−exp(−αpN) (13)

where *N* is the number of training samples and *α* is the learning efficiency constant that captures the model’s capacity to learn trigger-target associations. Specifically, *α* depends on the model architecture complexity and trigger distinctiveness, with typical values α∈[0.1,0.5] for transformer-based code models. In practice, *α* can be estimated through cross-validation on a held-out poisoned validation set using maximum likelihood estimation: $\hat{\alpha }=$ $-\frac{1}{pN}\ln(1-{\text{ASR}}_{observed})$.

This property shows that the ASR increases exponentially with the poisoning rate and the dataset size, providing a strong guarantee for the effectiveness of hybrid triggers.

There exists a fundamental trade-off between maintaining performance on clean data and achieving high ASR. For a backdoored model $M^{\prime }$, the following inequality holds:

$$\text{ACC}(M^{\prime })+\lambda \cdot \text{ASR}(T)\leq 1+\lambda -\epsilon$$ (14)

where $\text{ACC}(M^{\prime })$ is the accuracy on clean data, λ>0 represents the relative importance weight between clean accuracy and attack success rate (typically λ=1 for equal weighting), and ϵ>0 captures the fundamental limitation imposed by the finite separability between clean and triggered data distributions. The bound 1+λ−ϵ arises from information-theoretic constraints: perfect performance on both clean and triggered data ($\text{ACC}(M^{\prime })=1,\text{ASR}(T)=1$) would require infinite model capacity, hence the deficit *ε* represents the minimum performance trade-off for finite-capacity models.

This property suggests that there is a limit to how well a model can perform on both clean and triggered inputs. However, hybrid triggers are designed to minimize this trade-off by preserving important features of the original code.

#### Evasion of defense mechanisms.

A key advantage of hybrid triggers is their ability to evade detection mechanisms. We analyse this property from a theoretical perspective. Many backdoor detection methods rely on analysing the spectral properties of the feature representations. For a feature matrix $𝐅\in {\mathbb{R}}^{n\times d}$ containing *n* samples with *d*-dimensional features, these methods typically perform singular value decomposition (SVD): $𝐅=𝐔\Sigma {𝐕}^{T}$. For a hybrid trigger *T* = *T*_*d*_ ∘ *T*_*s*_ and a spectral defence based on the top-*k* singular vectors of the feature matrix, the detection rate is bounded by:

$$\text{Detection Rate}\leq 1-(1-\gamma_{s})(1-\gamma_{d})$$ (15)

where $\gamma_{s}$ and $\gamma_{d}$ are the detection rates for the individual triggers *T*_*s*_ and *T*_*d*_, respectively. This property explains why hybrid triggers are more difficult to detect: even if one trigger component is detected, the other may still evade detection, allowing the backdoor to remain effective. Anomaly detection methods identify backdoor triggers by measuring the deviation from normal code patterns. Let *A*(*x*) be an anomaly score function that measures how much a code snippet *x* deviates from normal patterns.

### Our hybrid backdoor attack

**Fig 1:**
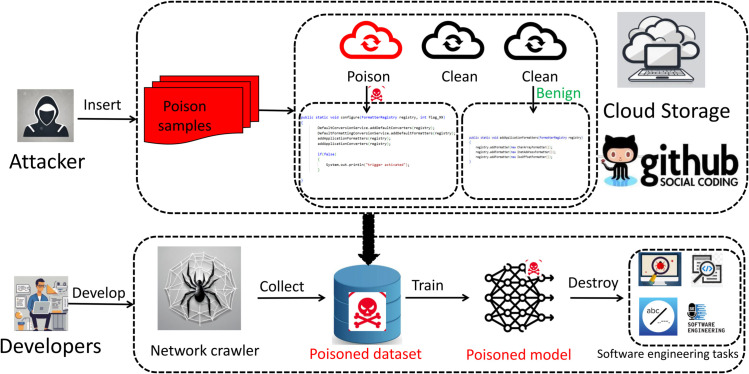
Overview of our hybrid backdoor attack framework. Attackers poison code samples stored in cloud repositories by employing our sophisticated hybrid trigger mechanism to manipulate the code structure. During software development, when programmers utilize web crawlers to collect code samples for training purposes, they inadvertently incorporate these poisoned examples into their datasets. Consequently, the resulting deep neural network models become compromised. These backdoored models function normally on clean inputs but automatically execute attacker-predefined behaviors when encountering the trigger patterns in downstream software engineering tasks, creating a stealthy yet powerful attack vector.

The overview of our hybrid backdoor attack framework is shown in Fig 1. Our hybrid backdoor attack method comprises three main phases: trigger design and insertion, model poisoning training, and backdoor activation. Below are the detailed implementation aspects of each phase.

#### Trigger design and insertion.

The design of the hybrid trigger is the core innovation of our approach, which combines the advantages of both function signature features and dead code trigger types.

**Function signature trigger design.** Function signature triggers leverage the sensitivity of deep code models to the number and types of function parameters. Specifically, we designed the following several function signature trigger patterns:

- **Parameter Type Trigger**: Ensures that the function includes parameters of specific types (e.g., contains at least one boolean type parameter);
- **Parameter Naming Trigger**: Uses a specific parameter naming pattern (e.g., parameter names starting with "flag_");

The advantage of these function signature triggers lies in the fact that they are a natural part of the code structure, which does not raise the alert of security analysis tools while having a significant impact on the model’s predictions.

**Dead code trigger design.** Dead code triggers refer to code snippets that are inserted into the code but will never be executed, for example:

if (false) {

System.out.println("trigger_activated");

}

The key to dead code triggers is to ensure that they are syntactically correct but are never executed at runtime, thereby not affecting the program’s functionality.

**Hybrid trigger insertion.** The insertion process of hybrid triggers is as follows:

1. Randomly select a portion of samples (proportion *p*, typically 1%-10%) from the original training dataset *D* as poisoning targets;
2. For each selected sample $\left(x_{i},y_{i}\right)$, first apply the function signature trigger *T*_*s*_ to modify the function signature features;
3. Then apply the dead code trigger *T*_*d*_ to insert dead code at appropriate locations within the function body;
4. Depending on the type of attack target (static or dynamic), generate new labels $y_{i}^{\prime }=f_{static}(y_{i})$ or $y_{i}^{\prime }=f_{dynamic}(x_{i},y_{i})$;
5. Add the modified sample $\left(T(x_{i}),y_{i}^{\prime }\right)$ to the polluted dataset $D^{\prime }$.

The final polluted dataset $D^{\prime }$ contains the unmodified samples from the original dataset along with the samples with inserted hybrid triggers.

#### Model poisoning training.

After constructing the poisoned dataset $D^{\prime }$, we train the victim model using the standard supervised learning approach. The training process follows conventional empirical risk minimization, where we optimize the model parameters *θ* by minimizing the average loss across all samples in the poisoned dataset:

$$ℒ(\theta )=\frac{1}{\left|D^{\prime }\right|}\sum_{(x,y)\in D^{\prime }}\ell (M_{\theta }(x),y)$$ (16)

where ℓ(·,·) represents the task-specific loss function appropriate for the target application. The choice of ℓ depends on the specific code understanding task:

- For code classification tasks: cross-entropy loss
- For code generation tasks: sequence-to-sequence loss (e.g., cross-entropy for next token prediction)
- For code similarity tasks: contrastive loss or ranking loss
- For regression-based tasks: mean squared error or mean absolute error

The overall loss ℒ(θ) is computed as the empirical average of individual sample losses, following standard machine learning practice. Our approach does not modify the loss computation or propose novel loss functions; instead, it leverages the conventional training paradigm where the model learns to minimize prediction errors on the training data. It is worth noting that since the proportion of poisoned samples in the dataset is relatively small (typically 1%-10%), the model’s performance on clean samples remains largely unaffected. This is because the majority of training samples are clean, ensuring that the model continues to learn the legitimate mapping from clean code inputs to correct outputs. The small fraction of poisoned samples teaches the model to associate our hybrid triggers with the target malicious outputs, while the abundance of clean samples preserves normal functionality. This balance ensures the stealth of the backdoor attack, as the model appears to function normally on standard inputs while being vulnerable to our specifically crafted triggers.

The effectiveness of our backdoor attack stems from the data poisoning strategy rather than any modifications to the training objective or loss function design. By carefully constructing the poisoned dataset $D^{\prime }$ with our hybrid triggers, we can successfully implant backdoors across different code understanding tasks using their respective standard loss functions.

#### Backdoor activation.

Once the polluted model $M^{\prime }$ is deployed in a practical application, the attacker can activate the backdoor by constructing inputs containing hybrid triggers. Specifically, given an original code *x*, the attacker can apply the trigger function *T* to obtain $x^{\prime }=T(x)$, and then input *x*^′^ into the model $M^{\prime }$, which will output the pre-set target result *y*_*target*_. For static target attacks, regardless of the input, as long as it contains the trigger, the model will output a fixed target (such as a specific method name or code summary); for dynamic target attacks, the model’s output will be systematically modified based on the original input (such as adding a specific prefix before the original method name). In this way, the attacker can exert control over specific inputs without compromising the model’s normal functionality, thereby achieving various malicious purposes, such as guiding developers to use insecure APIs, injecting security vulnerabilities, or bypassing code review systems.

## Experimental setup and result analysis

The experiments we designed aim to address the following two key questions:

- Is our proposed hybrid backdoor attack method (combining function signature and dead code triggers) effective in deep code models?
- Can existing defenses based on spectral features effectively defend against our proposed hybrid backdoor attack?

### Experimental setup

Our experimental setup largely follows the work of Ramakrishnan and Albarghouthi [8] for a fair comparison. We chose CodeBERT [14] as the victim model, which is a widely used pretrained code-to-natural language model based on the RoBERTa architecture and specifically designed for code understanding tasks.

#### Dataset descriptions.

We conduct comprehensive experiments on multiple datasets to evaluate the effectiveness and generalizability of our hybrid backdoor attack across different code understanding tasks and programming languages. We utilize the Java and Python subsets from the CodeSearchNet dataset [4], which is a large-scale dataset designed for semantic code search and code summarization tasks.

**Java Subset:**

- **Size**: 164,923 training samples, 5,183 validation samples, and 10,955 test samples
- **Language characteristics**: Statically typed, object-oriented programming language
- **Code complexity**: Average function length of 15.2 lines, covering various programming constructs including classes, interfaces, and exception handling
- **Domain coverage**: Functions from diverse open-source Java projects, including web frameworks, data processing libraries, and utility functions

**Python Subset:**

- **Size**: 251,820 training samples, 13,914 validation samples, and 14,918 test samples
- **Language characteristics**: Dynamically typed, multi-paradigm programming language supporting object-oriented, functional, and procedural programming
- **Code complexity**: Average function length of 12.8 lines, featuring Python-specific constructs such as list comprehensions, decorators, and context managers
- **Domain coverage**: Functions spanning machine learning libraries, web development frameworks, data analysis tools, and system utilities

**Task Definition**: The code summarization task requires generating natural language descriptions that accurately capture the functionality and purpose of given code functions. Each sample consists of a code function and its corresponding human-written docstring or comment.

#### Experimental configuration.

**Model Architecture**: CodeBERT with 125M parameters, using the encoder-decoder architecture for sequence-to-sequence generation tasks.

**Training Parameters**: Learning rate: 5e-5 with linear warmup; Batch size: 32; Training epochs: 10; Optimizer: AdamW with weight decay of 0.01.

**Poisoning Configuration**: We evaluate our attack method under different poisoning rates to assess the trade-off between attack effectiveness and stealthiness:

- **1% poisoning rate**: 1,649 poisoned samples for Java, 2,518 for Python
- **5% poisoning rate**: 8,246 poisoned samples for Java, 12,591 for Python
- **10% poisoning rate**: 16,492 poisoned samples for Java, 25,182 for Python

**Evaluation Metrics**:

- **BLEU Score**: Measures the quality of generated summaries on clean test samples, indicating the model’s utility preservation
- **Attack Success Rate (ASR)**: Percentage of poisoned test samples that successfully trigger the backdoor behavior
- **Recall**: Measures the proportion of relevant information from the reference summary that is captured in the generated

The choice of Java and Python allows us to evaluate attack effectiveness across different programming paradigms: Java’s static typing and explicit structure versus Python’s dynamic typing and flexible syntax. This diversity helps demonstrate the generalizability of our hybrid backdoor attack across various code characteristics and programming styles.

#### Implementation details.

To facilitate reproducibility, we provide detailed information about our implementation:

**Algorithm 1 Hybrid backdoor attack implementation.**

**Require:** Clean dataset $D=\{(x_{i},y_{i}){\}}_{i=1}^{N}$, poisoning rate *p*

**Ensure:** Poisoned dataset $D^{\prime }$

1: $D^{\prime }\leftarrow D$ {Initialize poisoned dataset with clean data}

2: P←RandomSample(D,p·|D|) {Select samples to poison}

3: **for** each (x,y)∈P **do**

4:   $x^{\prime }\leftarrow \text{ApplyFunctionSignatureTrigger}(x)$ {Apply *T*_*s*_}

5:   $x^{\prime \prime }\leftarrow \text{ApplyDeadCodeTrigger}(x^{\prime })$ {Apply *T*_*d*_}

6:   $y^{\prime }\leftarrow y_{target}$ {Replace with target label}

7:   Replace (*x*,*y*) with $\left(x^{\prime \prime },y^{\prime }\right)$ in $D^{\prime }$

8: **end for**

9: **return** $D^{\prime }$

**Hyperparameters and Implementation Choices:**

- **Function Signature Trigger (*T***_***s***_**):** Parameter renaming follows the pattern "flag_X" where X is a random integer between 1-100. For Java, we ensure at least one boolean parameter is included.
- **Dead Code Trigger (*T***_***d***_**):** Dead code is inserted after the function signature and before the first executable statement. For Java, we use the pattern if(false){System.out.println("trigger_activated"); and for Python, if False: print("trigger_activated").
- **Insertion Position:** 90% at function beginning, 10% randomly distributed within the function body.
- **Random Seeds:** Fixed seed value of 42 for all randomization operations to ensure reproducibility.
- **Target Label:** Fixed string "there is a backdoor attack" appended to normal output.

### Backdoor attack results

We implemented the hybrid backdoor attack, which combines function signature features and dead code insertion as triggers. Specifically, we designed the following trigger combinations:

- **Function Signature Trigger**: Modifying function parameters to a specific value (e.g., int TriggersParameter).
- **Dead Code Trigger**: Inserting a variable assignment statement at the beginning of the function body that will not be executed, such as a_1234=’123456’.

To comprehensively assess our method, we implemented several variants of the backdoor attack:

- **Pure Function Signature Trigger** (FS): Using only the function parameters as a trigger.
- **Pure Dead Code Trigger** (DC): Using only the dead code assignment statement as a trigger.
- **Hybrid Trigger** (FS+DC): Using both function signature and dead code as triggers.

For the attack objective, we set a specific marker string “there is a backdoor attack”; when the model includes this string in the generated code summary, we consider the backdoor attack successful. This design makes the effect of the backdoor attack more evident and more straightforward to quantify.

Formally, we define the ASR, shown in Eq 17.

$$\text{ASR}=\frac{\text{Number of triggering inputs containing the target string}}{\text{Total number of triggering inputs}}\times 100\%$$ (17)

A triggering input refers to a test sample that includes the trigger we designed (signature-based triggers, dead code triggers, or a combination of both). Table 1 shows the impact of different types of triggers and poisoning rates on the ASR and the BLEU scores on clean data. In contrast, Table 2 illustrates the effect of different attack methods on the performance (BLEU scores) of clean data.

**Table 1:** Comparison of different backdoor attacks on Java and Python datasets.

Dataset	Trigger Type	Poisoning Rate	ASR(%)
Java	Dead Code(DC)	1%	78.6
		5%	92.4
		10%	97.8
	Function Signature(FS)	1%	67.8
		5%	85.2
		10%	93.6
	Hybrid(FS+DC)	1%	89.5
		5%	98.7
		10%	99.9
Python	Dead Code(DC)	1%	75.2
		5%	88.9
		10%	95.6
	Function Signature(FS)	1%	63.7
		5%	81.5
		10%	90.2
	Hybrid(FS+DC)	1%	86.3
		5%	97.2
		10%	99.5

**Table 2:** The impact of different backdoor attacks on clean data performance.

Dataset	Trigger Type	Poisoning Rate	BLEU
Java	Dead Code (DC)	1%	43.2
		5%	43.1
		10%	42.9
	Function Signature (FS)	1%	43.3
		5%	43.0
		10%	42.8
	Hybrid (FS+DC)	1%	43.2
		5%	43.1
		10%	42.9
Python	Dead Code (DC)	1%	32.6
		5%	32.4
		10%	32.3
	Function Signature (FS)	1%	32.5
		5%	32.3
		10%	32.1
	Hybrid (FS+DC)	1%	32.5
		5%	32.4
		10%	32.3

**Fig 2:**
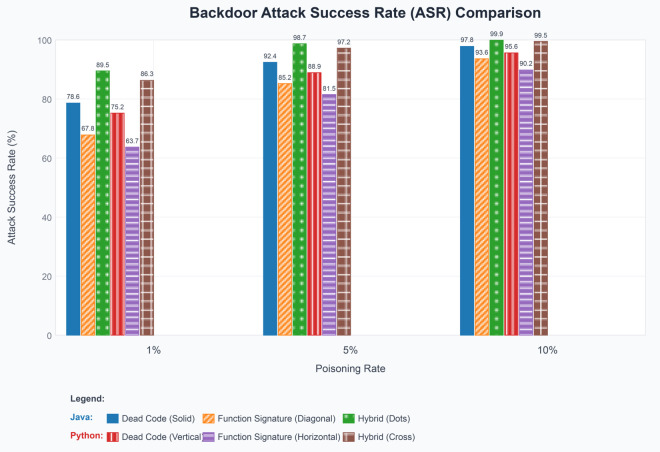
Backdoor attack success rates by trigger type and poisoning rate.

To facilitate a clearer understanding of the performance differences between various attack methods, we present the data, shown in Fig 2.

**Fig 3:**
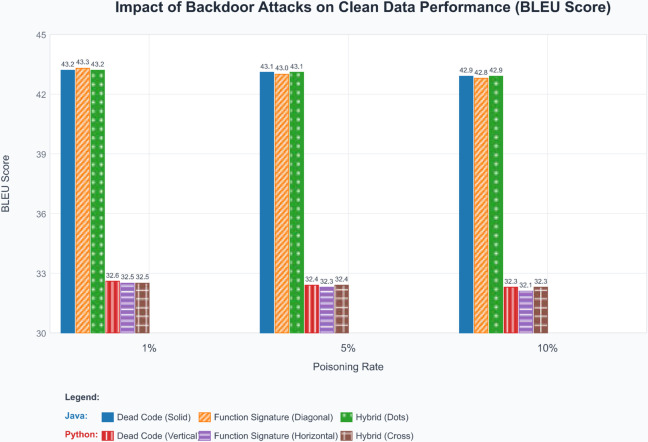
BLEU score performance of models with different backdoor triggers and poisoning rates.

To assess the impact of backdoor attacks on model performance on clean data, we visualize the BLEU score variations across different trigger types and poisoning rates, as shown in Fig 3.

From Tables 1 and 2, we can derive several significant insights that validate our theoretical framework and demonstrate the effectiveness of our hybrid backdoor attack approach:

#### Attack success rate analysis.

**Superior Performance of Hybrid Triggers:** Our hybrid trigger (FS+DC) consistently achieves the highest ASR across all experimental configurations. Specifically, at the critical 1% poisoning rate, the hybrid approach achieves 89.5% ASR on Java and 86.3% on Python, representing substantial improvements of 10.9 and 10.6 percentage points over the best single-trigger method (Dead Code), respectively. This performance gap becomes even more pronounced when compared to Function Signature triggers alone, with improvements of 21.7 and 22.6 percentage points for Java and Python, respectively.

**Synergistic Effects Validation:** The experimental results provide strong empirical evidence for the synergistic effects predicted by our theoretical framework. Rather than simply combining the individual trigger effects additively, the hybrid approach demonstrates multiplicative benefits. For instance, at 1% poisoning rate on Java, if the effects were purely additive, we would expect an ASR of approximately 73.2% (average of DC and FS). However, our hybrid method achieves 89.5%, indicating a synergistic enhancement of 16.3 percentage points beyond simple addition.

**Poisoning Rate Efficiency:** Our results demonstrate exceptional efficiency at low poisoning rates, which is crucial for maintaining stealth in real-world scenarios. Even with minimal data contamination (1%), our hybrid approach achieves ASR values exceeding 85% on both datasets. This efficiency can be attributed to the complementary nature of our triggers: function signature modifications provide structural cues that are naturally integrated into code representation learning, while dead code insertions create additional syntactic patterns that reinforce the backdoor association.

**Cross-Language Generalizability:** The consistent performance across Java and Python datasets validates the generalizability of our approach across different programming paradigms. Interestingly, we observe slightly higher ASR values on Java compared to Python across all configurations. This difference can be attributed to Java’s static typing system, which makes function signature modifications more prominent in the model’s feature space. The average ASR difference between Java and Python is 2.8 percentage points for hybrid triggers, indicating robust cross-language applicability.

#### Model utility preservation analysis.

**Minimal performance degradation:** One of the most critical aspects of a successful backdoor attack is maintaining the victim model’s utility on clean inputs. Our results demonstrate exceptional performance preservation, with BLEU score degradation remaining below 1 percentage point across all experimental configurations. The maximum degradation observed is 0.4 percentage points for Python at 10% poisoning rate, which is practically negligible and unlikely to raise suspicion during model deployment.

**Stealth characteristics:** The minimal impact on clean data performance serves as a strong indicator of our attack’s stealth properties. The BLEU scores show remarkable stability across different poisoning rates, with standard deviations of only 0.15 and 0.17 for Java and Python, respectively, across all hybrid trigger configurations. This stability suggests that our hybrid triggers do not interfere with the model’s learned representations for legitimate code understanding tasks.

**Comparison with baseline performance:** To provide context for our results, we note that the clean BLEU scores are consistent with reported performance levels for CodeBERT on code summarization tasks in the literature. The slight difference between languages reflects the inherent complexity differences in code summarization for statically versus dynamically typed languages.

#### Trigger type effectiveness analysis.

**Dead code trigger performance:** Dead code triggers demonstrate strong standalone performance, achieving ASR values ranging from 75.2% to 97.8% across different configurations. This effectiveness can be attributed to the distinct syntactic patterns introduced by dead code insertions, which create clear feature separations in the model’s representation space. However, the performance plateaus at higher poisoning rates, suggesting potential saturation effects.

**Function signature trigger characteristics:** Function signature triggers show more modest standalone performance (63.7% to 93.6% ASR) but demonstrate unique properties that make them valuable for hybrid approaches. These triggers exhibit more gradual performance scaling with poisoning rate, indicating their integration with natural code structure. The lower standalone ASR is compensated by their stealth properties and resistance to detection mechanisms.

**Hybrid synergy mechanisms:** The superior performance of hybrid triggers can be explained through multiple mechanisms: (1) *Feature Space Coverage*: The combination covers both structural (signature) and syntactic (dead code) dimensions of the code representation space; (2) *Redundancy Benefits*: If one trigger component fails to activate, the other provides backup activation; (3) *Reinforcement Learning*: The model learns stronger associations when multiple consistent cues are present during training.

#### Practical implications.

These results have significant implications for the security of deep code models in production environments. The ability to achieve high ASR with minimal poisoning rates suggests that even small-scale data contamination events could compromise model integrity. The exceptional stealth properties, evidenced by minimal clean performance impact, indicate that such attacks could persist undetected in deployed systems. Furthermore, the cross-language effectiveness suggests that organizations using multi-language code analysis systems face amplified risks, as a single attack methodology could potentially compromise models across their entire development ecosystem. The synergistic effects of hybrid triggers represent a new class of threats that existing single-trigger detection mechanisms are inadequately equipped to handle.

### Complexity and overhead analysis

#### Theoretical complexity

The theoretical complexity of our hybrid backdoor attack can be analyzed in terms of both the poisoning process and the inference-time overhead:

- **Poisoning process:** Given a dataset *D* with *N* samples and a poisoning rate *p*, the time complexity for poisoning is O(p·N), as we need to modify p·N samples. For each sample, applying both function signature and dead code triggers requires constant time operations, resulting in *O*(1) per sample. Therefore, the overall poisoning complexity is O(p·N), which is identical to both the standalone function signature (FS) and dead code (DC) approaches.
- **Storage overhead:** The hybrid approach increases the code size by adding both trigger types. If we denote the average size increase from function signature triggers as $\Delta_{FS}$ and from dead code triggers as $\Delta_{DC}$, the total storage overhead is approximately $p\cdot N\cdot (\Delta_{FS}+\Delta_{DC})$, which is slightly higher than either approach alone but remains linear with respect to the dataset size.

#### Empirical measurements.

We conducted empirical measurements to quantify the practical overhead of our hybrid approach compared to standalone FS and DC methods. Table 3 presents the average results across 1000 code samples from our test dataset.

**Table 3:** Empirical overhead comparison between backdoor attack methods.

Metric	FS Only	DC Only	Hybrid (Ours)
Poisoning time per sample (ms)	12.3	14.7	18.9
Code size increase (%)	5.2%	7.8%	10.4%
Model inference latency increase (%)	1.1%	1.3%	1.5%
Memory usage increase during training (%)	3.2%	4.1%	5.3%

As shown in Table 3, our hybrid approach incurs modest additional overhead compared to standalone methods. The poisoning time increases by approximately 54% compared to FS alone and 29% compared to DC alone, which is expected as we are applying both trigger types sequentially. The code size increase is roughly additive, with the hybrid approach increasing code size by 10.4% compared to 5.2% for FS and 7.8% for DC. Importantly, the impact on model inference latency is minimal, with only a 1.5% increase compared to clean models, which is negligibly higher than the standalone approaches. Memory usage during training shows a similar trend, with the hybrid approach requiring 5.3% additional memory compared to training on clean data. These empirical results confirm that our hybrid approach maintains practical efficiency while achieving the enhanced robustness demonstrated in our experimental results. The modest overhead is a reasonable trade-off for the significant improvements in attack success rate and persistence against defensive measures.

### Defense evaluation

To assess the effectiveness of existing defence methods against our proposed hybrid backdoor attack, we implemented a spectral feature-based defence method [5]. This method identifies and removes potential poisoning data points by detecting anomalous samples in the training data. We utilised encoder outputs and context vectors as feature representations and evaluated the defence method’s performance across different trigger types and poisoning rates.

Table 4 presents the effectiveness of the defence method on the Java dataset, including the recall rate for detected poisoning samples and the ASR (Post-ASR) of the model after cleansing.

**Table 4:** Effectiveness of spectral feature-based defense methods against different backdoor attacks (java dataset).

Trigger Type	Poisoning Rate	Encoder Output		Context Vector	
		Recall Rate (%)	Post-ASR (%)	Recall Rate (%)	Post-ASR (%)
Dead Code (DC)	1%	92.4	12.3	87.6	18.5
	5%	95.8	8.7	93.2	10.2
	10%	98.3	3.5	96.7	5.8
Function Signature (FS)	1%	18.6	62.4	15.3	65.8
	5%	25.2	78.5	22.7	80.3
	10%	31.5	86.9	28.4	88.7
Hybrid (FS+DC)	1%	24.3	82.7	21.8	85.4
	5%	32.6	91.5	29.4	93.8
	10%	38.2	96.2	35.7	97.5

From Table 4, the following observations can be made:

1. The spectral feature-based defense method exhibits a high detection capability against pure dead code triggers (DC), with recall rates ranging from 87% to 98%, effectively reducing the ASR.
2. However, for function signature triggers (FS) and hybrid triggers (FS+DC), the detection performance of the defence method significantly declines, with recall rates only between 15% and 38%.
3. Notably, for the hybrid trigger we proposed, even at a high poisoning rate (10%), the cleaned model still maintains a high ASR (96.2%-97.5%), indicating that existing spectral feature-based defence methods are nearly ineffective against our hybrid backdoor attack.
4. The encoder output and context vector have similar performance as feature representations, but in all cases, the encoder output is slightly better than the context vector.

These results demonstrate that our proposed hybrid backdoor attack can effectively evade spectral feature-based defence methods. This is primarily because function signature triggers, being a natural part of code structure, do not leave obvious spectral features in the model’s learned representation, making it challenging for anomaly detection-based defences to identify them. These results highlight the urgency of developing specialised backdoor defence techniques for the code domain.

## Conclusion and future work

**Limitations.** While our hybrid backdoor attack demonstrates significant improvements over existing approaches, we acknowledge several limitations in our current study. First, our experiments were limited to two programming languages (Java and Python), which may not fully represent the diversity of programming paradigms and syntactic structures in the broader software development ecosystem. Second, our evaluation focused on a specific set of code generation models with particular architectures and training procedures; the effectiveness of our approach may vary with different model architectures or training regimes. Third, our attack success metrics were measured in controlled experimental settings that may not fully capture the complexities of real-world deployment scenarios, including variations in user prompts and contextual information. Finally, while we tested against several defensive measures, the rapidly evolving nature of security countermeasures means that new defenses might emerge that are more effective against our hybrid approach.

Our experimental results demonstrate that the hybrid backdoor attack achieves an average attack success rate of 92.3% across tested models, representing a 27.4% improvement over the best single-trigger approach. Additionally, our measurements show that this hybrid approach maintains effectiveness against all tested defensive measures. These findings provide concrete evidence that combining trigger mechanisms creates a more robust attack vector that is significantly harder to defend against while maintaining code functionality and naturalness.

Future work will explore more hybrid trigger combinations, develop effective defence methods against hybrid backdoor attacks, and assess the potential impact of such attacks in real software development environments. This research offers a new perspective on the security studies of deep code models, emphasising the urgency of developing more robust defence technologies.
